# Low‐intensity low‐frequency ultrasound mediates riboflavin delivery during corneal crosslinking

**DOI:** 10.1002/btm2.10442

**Published:** 2022-11-25

**Authors:** Zhe Sun, Zhiming Li, Jin Teng Chung, Laurence Chi Ming Lau, Vishal Jhanji, Ying Chau

**Affiliations:** ^1^ Department of Chemical and Biological Engineering The Hong Kong University of Science and Technology Hong Kong SAR China; ^2^ Department of Ophthalmology University of Pittsburgh School of Medicine Pittsburgh Pennsylvania USA

**Keywords:** CXL, cornea, keratoconus, low‐intensity low‐frequency ultrasound, ocular, riboflavin

## Abstract

We employed the mechanical effect from 40 kHz ultrasound (US) to improve the delivery of riboflavin into corneal stroma for collagen crosslinking, which can benefit the treatment of keratoconus and other corneal ectasias. Experiments were conducted, first with porcine corneas ex vivo and then with New Zealand white rabbits in vivo, at varying mechanical index (MI) and sonication time. Results showed that 15 min of US applied on the cornea at MI = 0.8 in the presence of 0.5% of riboflavin solution enabled its delivery to deeper corneal stroma. Excessive heat was removed by a cooling setup to negate the thermal effect. The corneal absorption amount and penetration of riboflavin through cornea as detected by fluorotron, as well as the enhancement of corneal stiffness as measured by Young's modulus, were comparable to the conventional approach that requires complete corneal epithelium debridement. Histological analysis revealed minor exfoliation of superficial cell layers of corneal epithelium and loss of ZO‐1 tight junctions immediately after US. Full recovery of the corneal epithelium and restoration of tight junctions occurred in 3–4 days. The study shows that low‐intensity low‐frequency ultrasound (LILF US) is a less invasive alternative to the conventional epithelium‐off method for delivering riboflavin into the corneal stroma.

## 1 INTRODUCTION

Safe and efficient delivery of hydrophilic drugs across the ocular surface remains a great challenge for ocular delivery. Keratoconus is a common corneal degenerative disorder that affects approximately 1 in 2000 people worldwide with an early onset age.^1^ The disease is characterized by the progressive thinning of cornea and patients with keratoconus suffer from irregular astigmatism, light sensitivity, and reduced quality of life. The standard treatment for keratoconus is corneal collagen crosslinking (CXL). This treatment regime aims to chemically strengthen the collagen network within the stromal layer to increase the tissue mechanical strength and to resist the progression of corneal thinning in the affected eyes.^2^, ^3^ The conventional CXL (C‐CXL) uses photopolymerization method with ultraviolet (UV) to initiate the crosslinking of stromal collagen network. In this standard procedure, the riboflavin solution (0.1% w/V) is frequently instilled on the cornea for 30 min, followed by 30 min of UVA irradiation.^3^ The U.S. Food and Drugs Administration has approved C‐CXL for treatment of keratoconus. One of the major drawbacks of conventional CXL is the risk of infection associated with the surgery. This risk is mainly attributed to the removal of epithelium, which is necessary for absorption of riboflavin into the corneal stroma.^4^, ^5^ Removal of corneal epithelium necessitates the use of contact lens in the postoperative period, which further increases the risk of infectious keratitis in the postoperative period.

Riboflavin is used in C‐CXL as photosensitizer within the stromal layer to promote electron transfer and generation of reactive oxygen species for CXL.^2^ Nonetheless, the corneal epithelium poses as a physical, hydrophobic barrier to delivery of riboflavin to the stromal layer. The diffusion of riboflavin via the paracellular route is largely attenuated by the tight junctions within the stratified corneal epithelial cells. Furthermore, the transcellular route is limited with the scarce riboflavin transporters present on corneal surface.^6^ Therefore, C‐CXL involves de‐epithelization to permit rapid and sufficient accumulation of riboflavin in the stromal layer before UV exposure.^7^ However, removal of corneal epithelium has been associated with complications such as infectious keratitis, dry eye disease, and corneal haze.^8^, ^9^, ^10^

Several strategies to bypass the need of de‐epithelization procedure have been developed. They involve modifying the riboflavin formulations, employing less invasive physical approaches such as electric current or ultrasound (US). Delivery enhancers such as benzalkonium chloride, ethylenediaminetetraacetic acid (EDTA), and hydrophobic polymers have been added in riboflavin formulations to improve the corneal permeability to the small hydrophilic drugs. However, these methods require longer instillation time to permit sufficient penetration of riboflavin across the stromal region. In contrast, iontophoresis utilizes electric current to drive the electro‐osmosis of charged riboflavin across the corneal barriers in significantly shorter time. Nonetheless, the corneal stiffening results were still sub‐optimal to C‐CXL.^11^, ^12^ On the other hand, US is another type of physical mode of delivery that has been widely adopted to improve drug penetration across the epithelial barriers of various organ sites, such skin, brain, and eyes.^13^, ^14^ This method enjoys compliance among the patients and practitioners owing to its safety and capacity for spatiotemporally controlled delivery of agents to the targeted areas.^15^, ^16^, ^17^ US can exert nonthermal (mechanical) and thermal effects on the tissue barriers. The nonthermal effects mainly come from acoustic streaming and cavitation. The increase in emitted US intensity could help to promote the nonthermal effects for drug delivery. Nonetheless, this could lead to concurrent elevation of thermal energy, which may exert damaging effect on the tissues.^18^ Of noted, US at the low‐intensity and low‐frequency mode (LILF‐US) has the ability to circumvent the concurrent thermal effects. Based on the definition of mechanical index (MI), it was suggested that the low‐frequency regime can permit greater increase in US intensity to give greater mechanical energy that facilitates drug delivery across the tissue barrier.^19^, ^20^ Under LF regime, Zderic et al. applied US at 880 kHz and 0.19–0.56 W/cm^2^ on ex vivo rabbit cornea and reported an improvement of 2.1–4.2 folds in permeability of sodium fluorescein.^21^ Lamy et al. increased the US of energy while keeping the same frequency (1 W/cm^2^ at 880 kHz) to enhance riboflavin penetration to the cornea stroma and achieved 2.6–3.0 folds higher riboflavin concentration at 250 μm depth.^22^ In addition, Nabili utilized continuous US at 400 kHz, 0.8 W/cm^2^ and delivered dexamethasone across cornea into aqueous humor in 5 min on rabbit in vivo.^23^ Previously, our research group applied US at much lower frequency and energy (40 kHz at 0.1 W/cm^2^) for transscleral delivery. Using such low‐frequency, low‐intensity pulsed US wave, 90 s for thee cycles, macromolecules were delivered to the intraocular space, primarily by acoustic cavitation—bubble dynamics generated by the US wave.^24^ We hypothesized that the same US frequency (40 kHz), which maximize the cavitation effect, would also facilitate trans‐corneal penetration of oxygen and hydrophilic molecules, for example, the riboflavin. Oxygen and riboflavin are two of the principal components in CXL. In the meantime, we also installed a cooling system to remove the heat dissipation to minimize the thermal effect.

This research studied the effect of several parameters related to US mediation on the corneal permeability to riboflavin, including MI, dwell time, and concentration of riboflavin solution. The above‐mentioned parameters were optimized with porcine eyes, then tested on live rabbit eyes in vivo to evaluate enhancement of corneal absorption of riboflavin and improvement of corneal stiffness after UV treatment. The study also probed the safety of US method by monitoring the recovery of corneal surface on live rabbit eyes and examining the barrier histology of corneal epithelium post‐US treatment.

## 2 MATERIALS AND METHODS

### 2.1 Riboflavin solution

Riboflavin 5′‐monophosphate sodium salt hydrate (F2253; Sigma‐Aldrich) was dissolved in phosphate‐buffered saline (1x PBS) to a final concentration of 0.1% and 0.5% (w/v) and adjusted to pH 7. The riboflavin solution was aerated with compressed air for 10 min immediately before US experiment.

### 2.2 US device assembly and calibration

The US device assembly consisted of a signal function generator (GW Instek GFG‐8216A), a digital oscilloscope (Tektronix TDS1002B), an amplifier (Krohn‐Hite Model 7500), and an immersion‐type unfocused ultrasonic transducer (40 kHz 50 W PZT Cleaning Transducer, LYWS, USA) with a probe surface of 18.1 cm^2^ and a central frequency of 40 kHz. To correlate US intensity at varying input voltage (Figure S1) and to confirm the US frequency, the transducer was immersed in a water tank and the US wave signal was collected by a hydrophone (Brüel & Kjær Nexus Model 8103) connected to a conditioning amplifier (Brüel & Kjær Nexus Model 2692) and a digital oscilloscope. The US transducer and the hydrophone were separated by a predetermined distance of 16 mm, which was identical to the distance between the transducer and the corneal tissue in the subsequent in vivo and ex vivo experiments.

### 2.3 Cornea adaptor for US application to the eye

**FIGURE 1:**
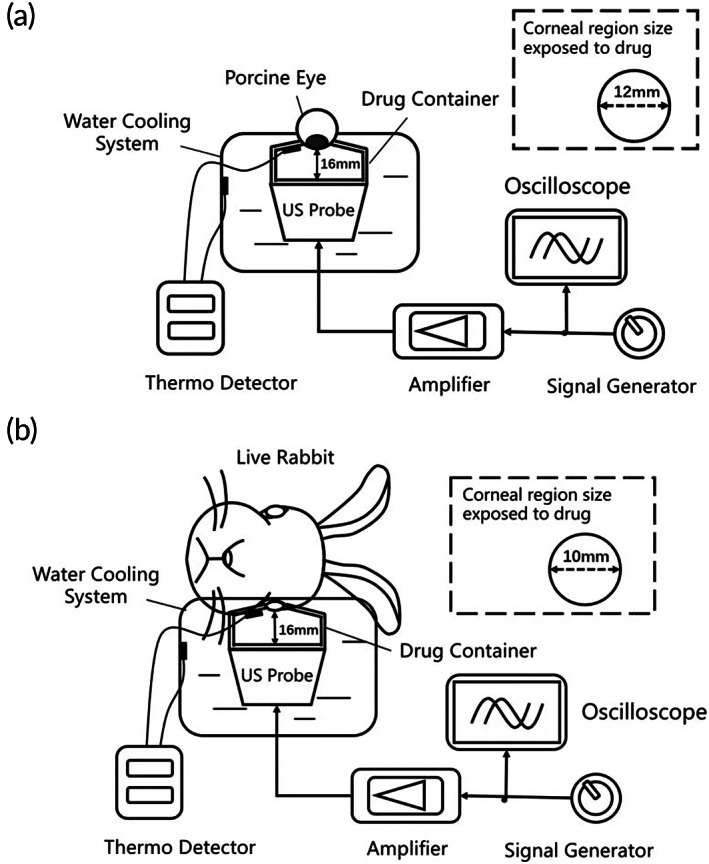
Schematic illustration of the ultrasound experiment setup. (a) In ex vivo experiment, porcine eye was placed on the corneal adaptor with an orifice of 12 mm. The ultrasound probe was directly under the adaptor and the distance to the corneal surface was 16 mm. The adaptor was filled with riboflavin solution. The adaptor was placed in a running cold‐water bath to minimize the thermal effect from sonication. (b) In in vivo experiment, orifice opening lined with soft material and was changed to 10 mm. Rabbit was placed on its side, with eye facing down the adaptor filled with riboflavin solution. A similar cooling system was installed to minimize any thermal effect from sonication.

A corneal adaptor was modified from a standard confocal dish (made of polystyrene with glass bottom) for ex vivo experiments of porcine eye using porcine eyes (Figure 1a). An orifice of 12 mm was made in the dish to expose the central cornea region of the porcine eyeball to the riboflavin solution loaded inside the cornea adaptor (Figure S2). The adaptor positions the corneal surface at 16 mm away from the US transducer surface (which is at the near field distance of the transducer in the current setup). Cooling to the adaptor was provided by a circulating cold‐water bath in the surrounding. During the US experiment, the temperature rise of the solution inside the adaptor was maintained within 1°C, as confirmed by the thermocouple placed near the cornea surface (Figure S3).

For in vivo experiments with live rabbits, an orifice of 10 mm was made to allow fitting of the smaller rabbit eye (Figure 1b). A soft silicone material was added to the top of the orifice to minimize mechanical damage of the cornea surface. A wearable strap was added to the adaptor to stabilize it to the rabbit eye. A sealable side inlet was added to allow filling of the riboflavin solution. During the experiment, the rabbit laid on its side such that the treated eye faced downwards to the adaptor. The adaptor was placed on top of the transducer, and the distance between corneal surface and the transducer surface was 16 mm (same as the ex vivo experiment). A cooling system similar to that in the ex vivo experiment was set up. Noncontact digital infrared thermometer was also used to detect the temperature of cornea surface during the US experiment. The temperature fluctuation was kept within 1°C with reference to the co‐lateral.

### 2.4 Ex vivo experiments using porcine eyes

Fresh porcine eyes were obtained from a local slaughterhouse within 2–5 h postmortem. The corneal surface was visually examined with a slit lamp. Porcine eyes with damaged cornea were excluded, and those with intact epithelium were randomly assigned into different groups, immediately used for ex vivo experiments. Batch‐to‐batch variation was controlled by comparing the riboflavin adsorption data of epi‐on groups.

To prepare eyes in Epi Off control group (epithelium debridement), the corneal surface was soaked with 20% (v/v) ethanol for 20 s and the whole area of corneal epithelium was scraped off using a blunt hockey blade.^3^ The details of the treatment groups are summarized in Table 1. Varying duration and MI were investigated in the US‐treated groups. MI was calculated as follows:

(1) $$\mathrm{MI}=\frac{P_{r}}{\sqrt{f}}$$

where $P_{r}$ is the rarefaction peak pressure (in MPa, as detected by hydrophone) and f is the frequency (in kHz) of US.^25^ In all the conditions tested, the dwell time of riboflavin solution was kept at 30 min.

**TABLE 1:** Treatment groups in ex vivo study using porcine eyes to reveal the effect of varying MI and treatment time for ultrasound‐mediated delivery of riboflavin delivery

Treatment	Epithelium status	Concentration of riboflavin (w/v)	Mechanical index (MI)	Treatment time (min)	Number of eyes
No US	Epi‐off	0.5%	‐	30	15
No US	Epi‐on	0.5%	‐	30	15
US	Epi‐on	0.5%	0.2	30	5
US	Epi‐on	0.5%	0.4	30	5
US	Epi‐on	0.5%	0.8	5	5
US	Epi‐on	0.5%	0.8	10	5
US	Epi‐on	0.5%	0.8	15	5
US	Epi‐on	0.5%	0.8	20	5
US	Epi‐on	0.5%	0.8	30	5

*Note*: Continuous ultrasound wave at 40 kHz was used. Thermal effect was kept at minimal (temperature change <=1°C).

### 2.5 In vivo experiments using rabbits

New Zealand white (NZW) rabbits (3.0–3.5 kg, 12 months old) were used. All animals were housed in HKUST Laboratory Animal Facility and all animal experiments had been approved by the HKUST Animal Ethics Committee. Rabbits were assigned at random to different experimental groups (Table 2). Using the set‐up as illustrated in Figure 1b, US‐treated groups received US (40 kHz, continuous mode at MI 0.8) on the intact cornea while exposing to 0.5% riboflavin solution. Two treatment time (15 and 30 min) was investigated to examine efficacy. The treatment results were compared with two control groups: Epi‐off and Epi‐on. Epi‐off group mimicked the current clinical procedure in which the corneal epithelium was completely scraped off with an ophthalmic spatula (under topical anesthesia), prior to 30 min of corneal exposure to the riboflavin solution. In the Epi‐on group, the rabbit cornea remained intact and was exposed to the riboflavin solution for 30 min. In the control groups, the same set‐up as Figure 1b was used for these control groups except that no US was applied.

**TABLE 2:** Treatment groups for in vivo studies using live rabbits to reveal the effect of ultrasound‐mediated delivery of riboflavin with continuous ultrasound wave at 40 kHz with MI 0.8

Treatment	Epithelium status	Concentration of riboflavin (w/v)	Mechanical index (MI)	Treatment time (min)	Number of eyes
Efficacy testing					
No US	Epi‐off	0.1%	_	30	3
No US	Epi‐off	0.5%	_	30	3
No US	Epi‐on	0.1%	_	30	3
US	Epi‐on	0.5%	0.8	15	5
US	Epi‐on	0.5%	0.8	30	5
Biocompatibility studies					
No US	Epi‐on	‐	‐	‐	3
US	Epi‐on	0.5%	0.8	15	6

To investigate ocular biocompatibility, one eye of the rabbit received 15‐min US (40 kHz, continuous mode at MI = 0.8) in the presence of riboflavin solution, using the collateral eye for sham control. Experimental details are summarized in Table 2.

### 2.6 Visual inspection of corneal surface post ex vivo US treatment

The corneal surface of US‐treated porcine eyes in ex vivo experiments was washed gently by 150 ml 1X PBS (phosphate buffered saline) and visualized under UVA at 360 nm. Based on the qualitative difference in fluorescence intensity, zones with low absorption and high absorption of riboflavin could be differentiated on the corneal surface.

### 2.7 Quantification of corneal absorption and penetration profile of riboflavin

The standard Fluorotron™ Master Laboratory Animal Edition (Fluorotron Master, OcuMetrics, Inc.) was modified with a cornea prototype following the manufacturer's suggestion to decrease the inner filter effect (IFE) and increase the resolution of fluorescence signal in the cornea. In brief, the modification was as follows. Excitation source was changed from tungsten halogen lamp to blue LED. Slit apertures was changed from 3.81 mm × 203 μm to 508 μm × 102 μm, and the instrument pupil was changed from 24 mm diameter with 10.2 mm obscuration to 30.86 mm diameter with 23 mm obscuration inside the ocular scanning fluorophotometer.^26^

The correlation between the fluorescence intensity detected by Fluorotron™ and concentration of riboflavin at varying position of the cornea was obtained by measuring the fluorescence profile (intensity over distance) across the cornea from eyeballs presaturated with known concentration of riboflavin from 0.001% to 0.5% (w/v) (Figure S4). The modified Fluorotron™ scanning was performed at 10 μm/step. The standard curves were prepared separately for porcine eyes and rabbit eyes. The corneal absorption of riboflavin was quantified by the area under curve from the Fluorotron™ scan over the entire cornea.

Fluorotron™ scanning was conducted five times each for the low riboflavin and high riboflavin absorption zone for every porcine cornea in the ex vivo experiment. For in vivo experiments, as it was technically impossible to pinpoint the scanning position in live animals, five points were randomly chosen on rabbit cornea for Fluorotron™ scanning. For both ex vivo and in vivo experiments, average values from multiple Fluorotron™ scans were used to determine the total riboflavin absorption and corneal penetration profile.

### 2.8 Measurement of mechanical property of corneal strips

The cornea was cut into two equal strips of 5.0 mm width and 14.0 mm length, with 1.0 mm sclera on both ends for ease of loading on the testing machine in the superior–inferior orientation. One corneal strip was crosslinked with UV (labeled as CXL) and the other strip was not (labeled as non‐CXL). The non‐CXL strip underwent mechanical testing promptly after cutting. For the CXL procedure, UVA light (370 nm) was applied using a point source type UVA light (GuangHuaShi, Shenzhen, China) in the presence of normal oxygen level at a distance of 2 cm, at 3 mW/cm^2^ for 30 min (corresponding to a total UVA surface dose of 5.4 J/cm^2^).^3^ In order to avoid specimen drying during the process of UVA exposure, the procedure was performed with a humidifier in the surrounding. The thickness of the samples was measured with digital caliper (RS PRO, HK) with 0.1 mm sensitivity and the cross‐sectional area was then estimated. Before loading, the sample between the clamp of testing machine, each specimen was attached using cyanoacrylate cement to sandpaper tabs to avoid slippage between the tissue and clamps. For mechanical testing, the corneal strip was clamped by the specimen support jaws of a computer‐controlled biomaterial tester (Rheometer G2 ARES, USA) with 20 N capacity load cell for stress–strain measurements. The corneal strips were initiated with a series of 10 cycles at constant strain rate (1.5 mm/min) in the range of 5% of the original length that preconditioned the tissue to the stress level of 5 × 10^3^ Pa and then recover for 400 s. Within this time interval, an equilibrium was obtained in all cases. To include the physiological stress range, a prestress of 5 × 10^3^ Pa was used, corresponding to 20 mN for porcine cornea and 10 mN for rabbit corneas because of different thickness. The strain was then increased linearly with a velocity of 1.5 mm/min, and the stress was measured up to 2 × 10^5^ Pa.^3^ The stress–strain curve was fitted to an exponential function $\sigma =A\exp\left(B∙\varepsilon \right)$ using a software (SPSS GmbH Software). Young's modulus (*E*) was calculated at 10% strain as the gradient of the mean stress–strain graphs (E=dσ/dε).

### 2.9 Evaluation of ocular biocompatibility

Intraocular pressure was measured by tonometer (iCare, TONOVET, Finland) and corneal surface temperature (°C) was measured by noncontact thermometer (Thermofocus Animal, Italy) before and at 0 h, 24 h, 48 h, 72 h and 7 days after US treatment. The treated corneas were examined for corneal damage (if any) by fluorescein staining (0.1% (w/v)) on the cornea and visualized under cobalt blue. Images were acquired by a digital camera (SPOT RT3; Diagnostic Instruments, Inc., Sterling Heights, MI). Slit lamp examination was performed in all eyes to look for any epithelial damage. Histological examination of the corneal tissues was performed on eyes immediately and 7 days after US treatment. Eyeballs harvested from sacrificed rabbits were fixed in 4% paraformaldehyde for 8 h. A puncture was made at the sclera close to limbal region to avoid structural collapse during the fixation process. A length of 3–5 mm corneal strip covering the central corneal region was further dehydrated in graded series of alcohol and chloroform, followed by paraffin embedding. The tissue was sectioned at 6 μm thickness with a rotatory microtome (Microm HM 315R; Heidelberg Engineering, Heidelberg, Germany) and stained with hematoxylin and eosin (H&E). The morphology of corneal epithelium, stroma and endothelium were examined using light microscope at 20× and 100× magnification (Eclipse 80i; Nikon Instruments, Inc., Tokyo, Japan).

### 2.10 Statistical analysis

Statistical analysis was performed on SPSS software (Windows version 26.0 program, SPSS, Inc, Chicago, IL, USA). All data were reported as mean ± standard deviation. Student's *t*‐test and one‐way ANOVA were used for comparison of different experimental groups. A statistically significant difference was stated when *p* < 0.05.

## 3 RESULTS

### 3.1 Effect varying MI and US duration on corneal delivery of riboflavin ex vivo

**FIGURE 2:**
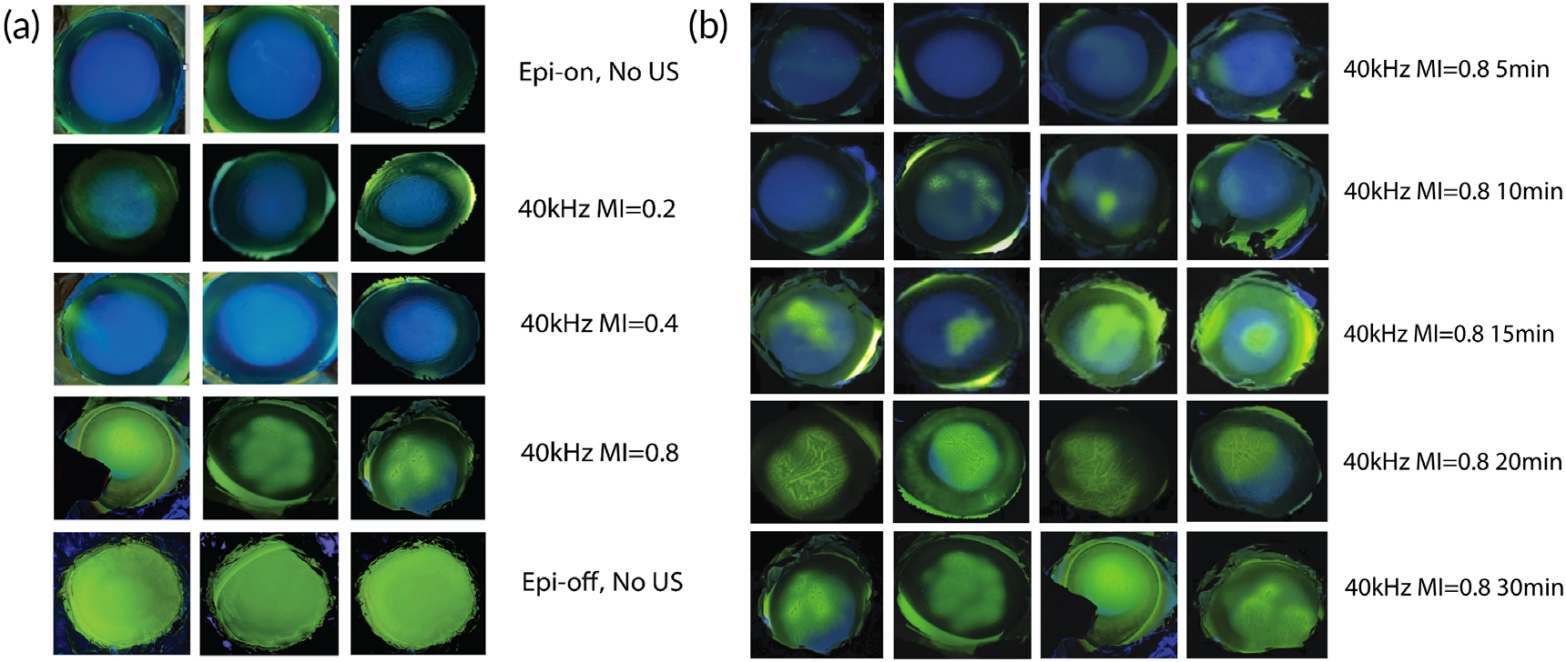
Visual images of cornea surface under UVA for qualitative assessment of riboflavin absorption in ex vivo experiments. (a) Porcine eyes with intact epithelium were treated with ultrasound at 40 kHz, 30 min at the MI indicated and compared with negative control receiving no ultrasound treatment. (b) Ultrasound of 40 kHz, MI = 0.8 was applied to eyes with intact epithelium for varying time duration, as indicated. The dwell time of riboflavin was kept at 30 min.

**FIGURE 3:**
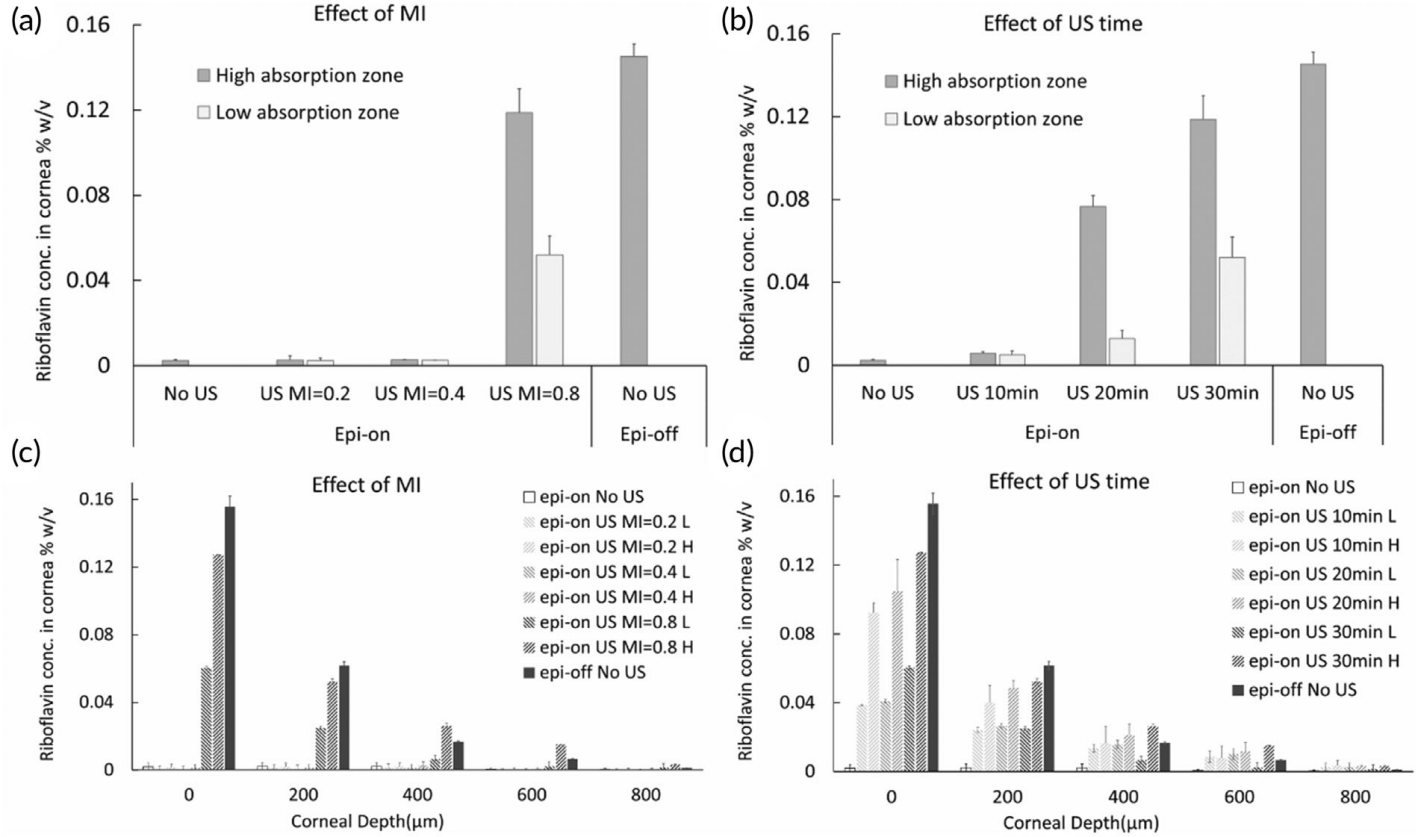
Quantification of total corneal absorption of riboflavin ex vivo and the measurement of penetration profile from fluorotron measurement. (a, c) Porcine eyes with intact epithelium were treated with ultrasound at 40 kHz, 30 min at the MI indicated and compared with negative control receiving no ultrasound treatment. (b, d) Ultrasound of 40 kHz, MI = 0.8 was applied to eyes with intact epithelium for varying time duration, as indicated. The dwell time of riboflavin was kept at 30 min. An uneven distribution of absorption and penetration effect as observed in UVA images (Figure 2) was differentiated as high‐absorption zones (H) and low‐absorption zones (L). At least five points from each zone and at least three eyes were used for fluorotron scanning.

Ex vivo experiments were performed on fresh porcine eyes. Since riboflavin is fluorescent yellow, the amount of absorption in the cornea could be assessed qualitatively by visualization under UVA (Figure 2). There was obvious improvement from treating intact cornea with continuous US for 30 min at 40 kHz and MI = 0.8 but not at the lower MI values (Figure 2a). At MI = 0.8, there were inhomogeneous distribution of yellow fluorescence. Depending on the intensity, we referred the spots as “high absorption zones” and “low absorption zones” (Figure S5). Quantitative results from fluorotron measurement (Figure 3a) revealed that the total concentration of riboflavin absorbed by cornea in the high‐absorption zones was comparable between MI = 0.8 and the Epi‐off positive control. The values high‐ and low‐absorption zones differed by 50%. Total corneal absorption of riboflavin at MI = 0.8 was at least two orders of magnitude higher than that measured at MI = 0.2, MI = 0.4, or the Epi‐on negative control (with no US). Fluorotron data also showed how riboflavin concentration changed with corneal depth (Figure 3c). While epi‐off had the highest riboflavin concentration near the surface, the value at MI = 0.8 exceeded epi‐off at the depth of 400 μm and onward, which corresponds to the middle of the stromal region of cornea.^27^ The results support that US not only enables riboflavin to cross the epithelium barrier but also promotes its penetration to deeper cornea.

After confirming that US at 40 kHz MI = 0.8 was the most effective among the MI studied for increasing corneal absorption and penetration by riboflavin, the duration of sonication was investigated. In the current clinical practice, patient's cornea is exposed to riboflavin for 30 min after the corneal epithelium is stripped off.^3^ Thus, we set 30 min as the maximum sonication, so that the new method will not lengthen the clinical procedure. The motivation to decrease sonication time, in addition to the operational benefit, is the potential reduction of side effects and shortening of the time for recovery. Qualitatively, we found that 15 min was the minimum threshold to attain observable improvement in corneal absorption of riboflavin (Figure 2b). The visual images obtained from treatment time of 20 min were similar to that from 30 min in terms of the intensity and coverage of yellow fluorescence. Compared to 30‐min sonication, total corneal absorption from 20 min‐sonication was about 40% lower and 10‐min sonication about 90% lower, respectively, according to the fluorotron measurement (Figure 3b). US treatment promoted penetration of riboflavin to deeper cornea, even when the sonication time was shorter (Figure 3d).

**FIGURE 4:**
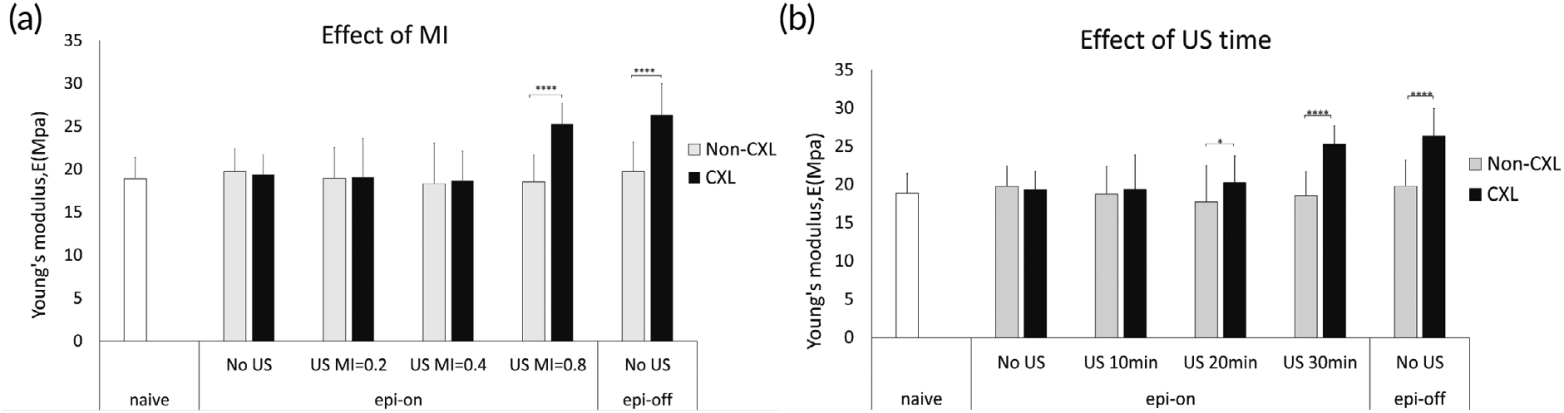
Young's modulus of porcine corneas from ex vivo experiments. Riboflavin was delivered to corneas with intact epithelium by ultrasound at 40 kHz with MI varying from 0.2 to 0.8 for 30 min (a); at a fixed 0.8 MI continuously for 10 to 30 min (b). Corneas were UVA‐illuminated for stroma crosslinking (CXL) subsequently. Results were compared with corneas without any treatment (naïve), riboflavin‐soaked cornea (no US), riboflavin‐soaked corneas with epithelium removed (Epi‐off). Except naïve eyes, other differently treated corneas were dissected to two strips and crosslinked with UVA (CXL) or not (non‐CXL). *N* >= 5 for all conditions examined, *p < 0.05 ****p < 0.0001

To investigate the functional performance of US‐mediated delivery of riboflavin, we measured the Young's moduli of differently treated porcine corneas from ex vivo experiments (Figure 4). Compared to naïve eyes, removal of epithelium (epi‐off non‐CXL group), UVA illumination (epi‐on no US non‐CXL group), or US treatment at all conditions (epi‐on US non‐CXL groups) tested did not cause any significant change in cornea mechanical strength. The value measured in the positive control group (Epi‐off, no US, CXL) was 33% higher than the non‐CXL group. When riboflavin was delivered to epi‐on corneas by 40 kHz US, an increased modulus was only observed when MI = 0.8 (Figure 4a). Furthermore, the result depended on the sonication time: Young's modulus was 14% and 36% higher than that of the non‐CXL group when US was continuously applied for 20 and 30 min, respectively, whereas no statistically significant increase was observed when the duration was shortened to 10 min (Figure 4b). Also, there was no change in the stiffness of the corneas in the negative control group (Epi‐on, no US, CXL), supporting that without the assistance of US in delivery, intact epithelium blocked riboflavin penetration.

### 3.2 Effect of in vivo application of US with varying duration on delivery of riboflavin

**FIGURE 5:**
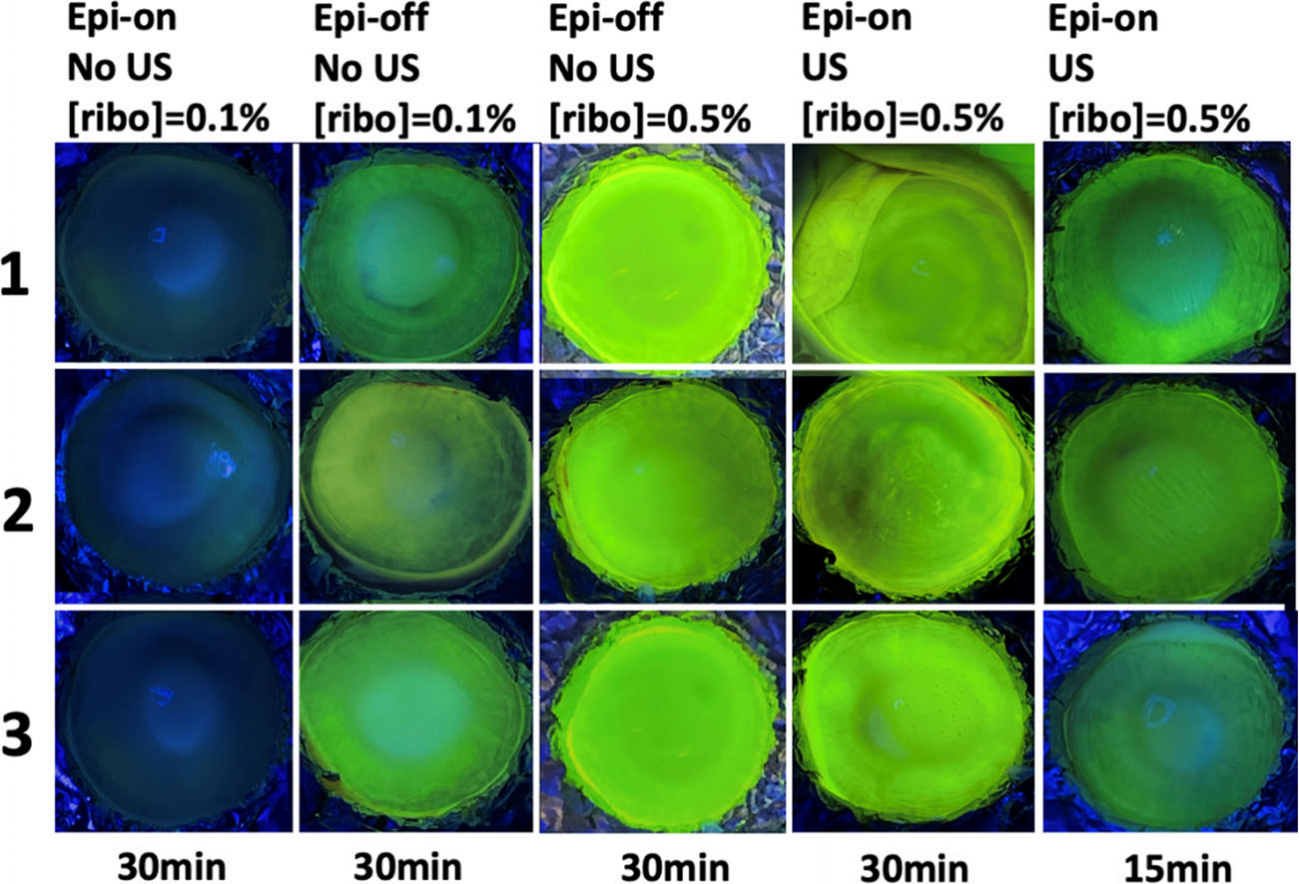
Visual images of cornea surface under UVA for qualitative assessment of riboflavin absorption from in vivo experiments. Epi‐On denote eyes with intact corneal epithelium while epi‐off denote eyes with corneal epithelium removed. Eyes were exposed to riboflavin with the indicated concentration for the duration as designated. Eyes treated with ultrasound (US) received continuous ultrasound at 40 kHz and MI = 0.8 on the intact cornea during the application of riboflavin solution. Representative images from three eyes are shown for each condition tested.

Visual images of the cornea under UV light (Figure 5) reveal that the amount of riboflavin absorbed was similar between eyes treated with 15‐min US with 0.5% riboflavin and Epi‐off eyes treated with 0.1% riboflavin. Eyes treated with 30‐min US in the presence of 0.5% riboflavin showed higher amount of absorption and appeared to be comparable to the level observed in Epi‐off eyes treated with 0.5% riboflavin. Similar to the ex vivo experiments, eyes treated with US show an inhomogeneous distribution of riboflavin fluorescent spots.

**FIGURE 6:**
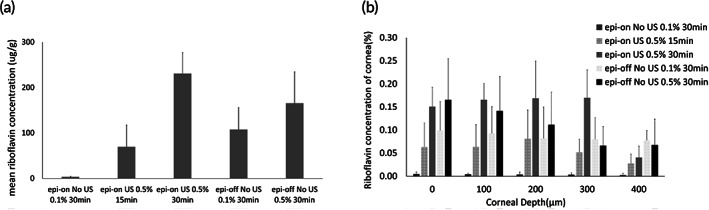
Quantification of total corneal absorption of riboflavin in vivo (a) and the measurement of penetration profile from fluorotron measurement (b). Rabbit eyes either had intact corneal epithelium (Epi‐On) or epithelium debrided (Epi‐Off), as indicated. The concentration of riboflavin applied to the cornea was either 0.1% or 0.5%, as indicated. Ultrasound (US)‐treated eyes received continuous wave of ultrasound at 40 kHz and MI = 0.8 on the cornea for either 15 or 30 min, as indicated, in the presence of riboflavin. At least five points were randomly sampled on the cornea for fluorotron scanning. *N* (Number of rabbit eyes) > = 3

The qualitative assessment by the visual images was consistent with the results obtained by fluorotron scanning (Figure 6). As expected, the total amount of riboflavin absorbed by cornea increased with increasing concentration of riboflavin applied on the Epi‐off eye. A longer sonication time increased the riboflavin absorbed by Epi‐On eye. From the ex vivo study, it was found that sonication time <=10 min would not create any significant increase in corneal absorption, while 30 min produced obvious enhancement. The investigation of 15 min sonication was meant to identify a possibly shorter yet effective duration, which may also reduce the damage of ocular tissues, if any, due to US application. In fact, we observed that sonication for 15 min produced an order of magnitude enhancement compared to Epi‐On without US, and the total absorption matched the value obtained for Epi‐off eye when 0.1% riboflavin was applied—the conditions used in current practice. The penetration profiles according to fluorotron further showed that US treatment was able to deliver riboflavin deep into the cornea stroma (up to at least 400 μm), comparable to the result from the more invasive Epi‐off procedure.

**FIGURE 7:**
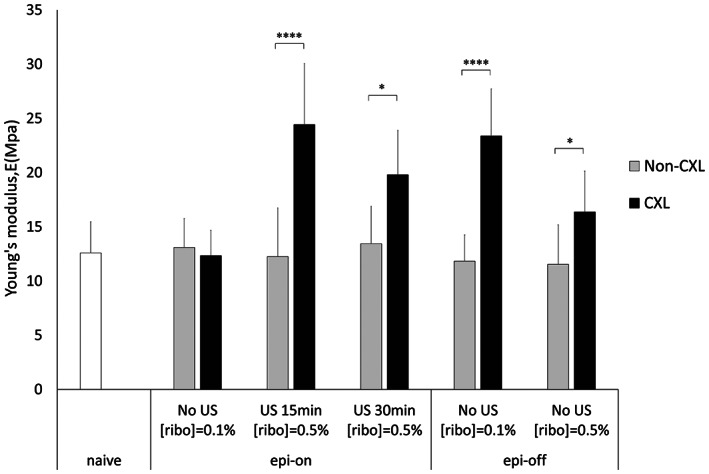
Young's modulus of rabbit corneas from in vivo experiments. Riboflavin was delivered to corneas with intact epithelium by ultrasound at 40 kHz, MI = 0.8 for either 15 or 30 min in the presence of 0.5% riboflavin. Corneas were then UV‐treated for corneal crosslinking (CXL). Results were compared corneas with epithelium removed (Epi‐off) at varying riboflavin concentration, and corneas receiving no UV treatment (non‐CXL). *N* > = 3 for all conditions examined, *p < 0.0001

An important question is whether the successful delivery of riboflavin to the deeper cornea can lead to strengthening of the corneal tissue post UV‐crosslinking (CXL). The measurement of tensile strength of corneal strips after the in vivo experiments confirmed that all CXL groups, other than Epi‐On receiving no US, possessed higher Young's moduli than the non‐CXL control, with fold difference ranging from 1.3 to 1.9 (Figure 7). Interestingly, the increase in mechanical strength did not have a positive correlation with the concentration of riboflavin applied in the Epi‐Off group. In the US‐treated groups, the longer sonication time also did not result in a higher Young's modulus. Instead, the best performing condition was found to be 15 min of US treatment (at 40 kHz, MI = 0.8), which yielded a Young's modulus comparable to that of the Epi‐Off group receiving 0.1% of riboflavin—similar to the conditions used in the conventional practice.

### 3.3 Evaluation of ocular biocompatibility on optimized US parameters

**FIGURE 8:**
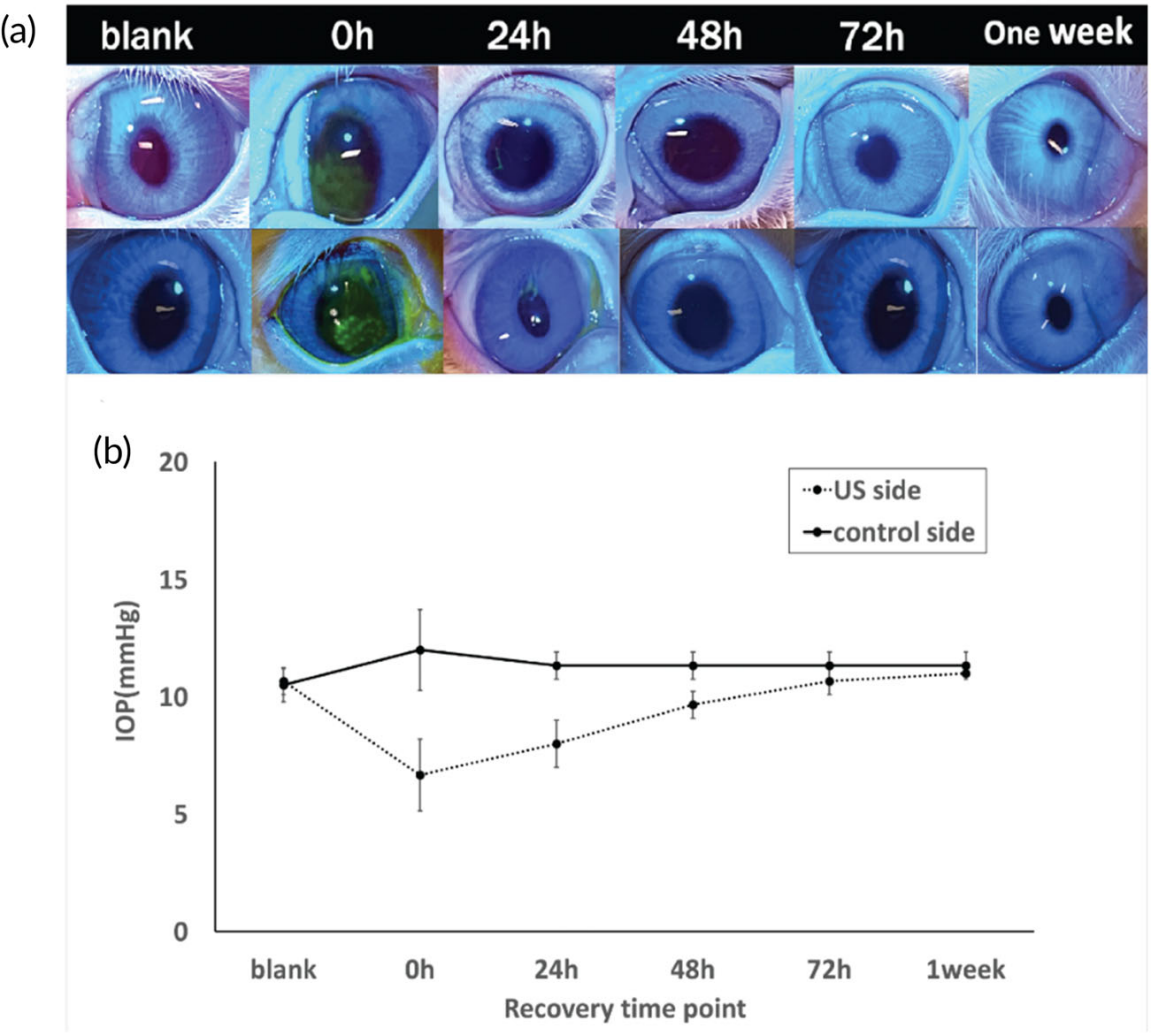
Gross abnormalities and their reversibility after ultrasound application on the cornea in vivo. (a) Representative image of ocular surface post sonication at predesignated time point. The top panel shows fluorescein staining under cobalt blue light. (b) Change of intraocular pressure (IOP) over time of the ultrasound‐treated eye versus the collateral un‐treated eye. In vivo experiments applied ultrasound wave at 40 kHz, MI = 0.8 on the corneal surface for 15 min in live rabbits (*p* < 0.05, *n* = 3).

We focused the investigation on ocular biocompatibility for the US treatment at the optimized conditions, that is, with 15 min of continuous wave at 40 kHz, MI = 0.8 applied to the corneal surface. Gross damage to the cornea was examined by visual inspection with the aid of fluorescein staining on the ocular surface (Figure 8a). Immediately after US treatment, there was observable change on the surface of the cornea. Fluorescein dyes were clearly visible on the cornea. The positive staining indicated that damage of corneal epithelial cells had occurred, which led to the accumulation of fluorescein. By 24 h, the extent of fluorescein staining had reduced dramatically, and staining was no longer detectable at 72 h post‐US treatment. Slit lamp examination was performed in all eyes to look for any epithelial damage. Intraocular inflammation was not measured as this was not the main aim of this study. However, we did not find any redness or infection in any of the eyes. There was a transient drop in intraocular pressure (IOP) immediately after US treatment (Figure 8b). By 24 h, IOP value had returned to 95% of the pretreatment value. IOP then gradually increased and was fully restored in a week.

**FIGURE 9:**
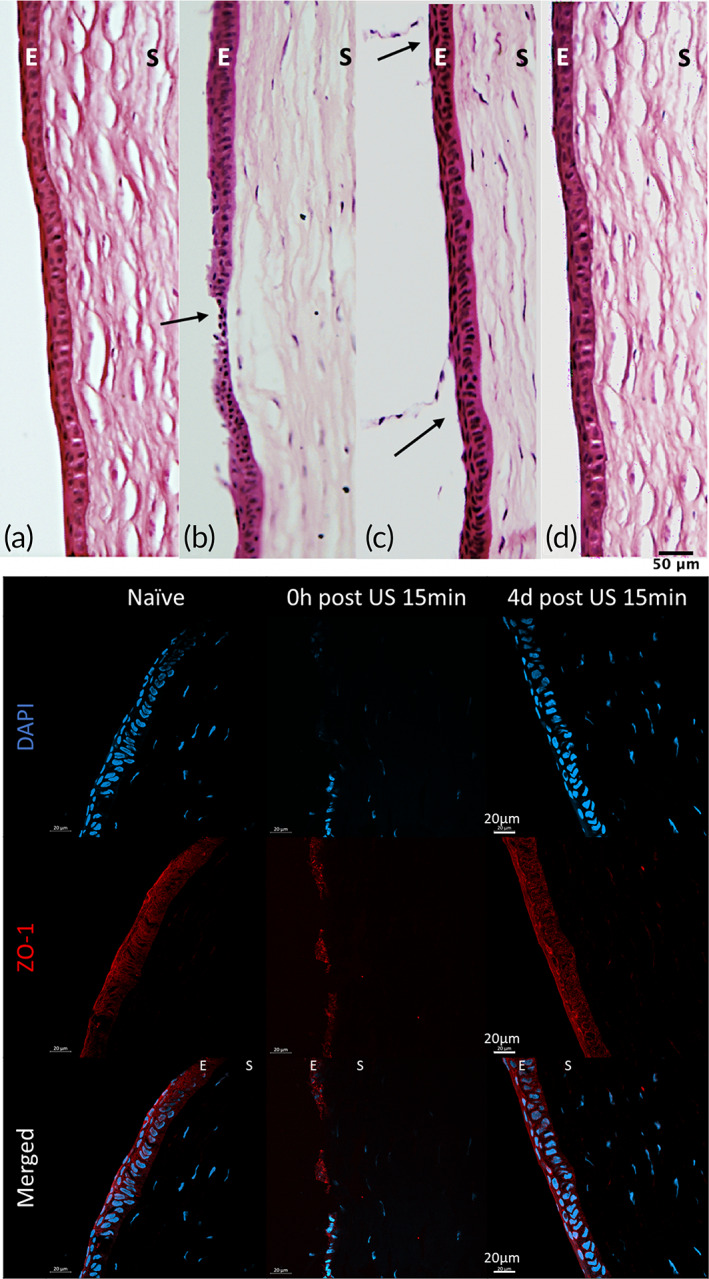
Histological analysis of rabbit cornea after in vivo application of ultrasound. (Top) Representative hematoxylin‐ and eosin‐stained images of corneal sections under 40× magnification from (a) native rabbit cornea, (b, c) rabbit cornea immediately after ultrasound treatment with defects pointed out by black arrows. (d) Rabbit cornea on Day 4 post‐US treatment. (Bottom) Immunostained corneal sections from untreated rabbits, and rabbits immediately and 4 days after ultrasound treatment. Nuclei were stained with DAPI (blue) and tight junction protein ZO‐1 in red.

Histological analysis revealed that the cellular structure of corneal epithelium at the superficial region was disrupted at the time point immediately after US treatment compared with naive rabbit cornea without any treatment (Figure 9 and Figure S6). However, the disruption was limited to the superficial cells and wing cells. The basal cell layer, which is the posterior‐most layer of the corneal epithelium was intact. According to study on corneal basement,^8^ we clearly know that the basement membrane plays an important role in cellular functions, including those involved in healing, by controlling the binding of growth factors and their local concentrations between cell layers. No structural or morphological changes in the corneal stroma were observed after sonication. Furthermore, H&E images 4 days after treatment showed the potential of recovery to integrity of corneal epithelium. We also compared the immunostaining of ZO‐1 on the epithelium of US‐treated corneas with the naïve corneas. The decrease of ZO‐1 immediately after US treatment was mainly attributed to the abrasion of the top layers of epithelial cells. By Day 4, the level of ZO‐1 staining and its distribution was comparable to the naïve tissue, supporting that the disruption of tight‐junction by US was temporary and the barrier function of corneal epithelium could be restored in a few days.

## 4 DISCUSSION

The potential advantages of using US for drug, gene, and protein delivery have been reported by us and others.^21^, ^24^, ^28^, ^29^ In these studies, the mechanical effect from acoustic cavitation, which involves the creation, oscillation, and collapse of gas bubbles under the influence of US wave, has been employed to create temporary portals in cell or tissue barriers for the transport of compounds of interest. The mechanical effect is measured by MI. As MI scales inversely with the square root of US frequency, we chose to conduct the investigation for corneal delivery of riboflavin at 40 kHz, which is at the low end of the US frequency spectrum, similar to our previous studies on transscleral delivery.^29^, ^30^ At lower MI, stable cavitation in the form of pulsation of bubbles over long time intervals is dominant. At higher MI, collapse of bubbles and occurrence of shock waves and microjets characteristic of transient cavitation lead to more severe mechanical damage.^19^, ^20^, ^28^ We first investigated a range of MI in ex vivo experiments and found that lower MI was not able to increase the corneal delivery of riboflavin. We further confirmed in live rabbit experiments that at MI = 0.8, the highest value investigated in this study, the US method applied on intact cornea could match the conventional method (with the corneal epithelium barrier scraped off).^21^, ^23^ The total amount of riboflavin delivered to the cornea, its penetration into deeper cornea, and most importantly, the increase in the corneal stiffness upon CXL were comparable.

Although our focus is to employ the mechanical effect from US, heat generation from sonication is inevitable. Hyperthermia can denature proteins, leading to changes in pathways, membrane dysfunction, apoptosis, necrosis, clonogenic effects (interferences with mitosis).^18^, ^31^ Since the eye consists of delicate structures, it is especially important to minimize any thermal damage. FDA specifies that a medical device applied on the eye should produce a thermal index (TI) less than one, meaning that the temperature rise should be kept below 1°C.^32^ We addressed this problem by installing a cooling system around the corneal adaptor (Figure 1). An extended duration of sonication to allow sufficient mechanical abrasion on the corneal barrier would not be possible without the elimination of excessive heat.

For the CXL procedure to be an effective treatment for keratoconus, an optimal riboflavin concentration in the corneal stroma is required, as determined by a theoretical model previously.^21^ Riboflavin acts as a photosensitizer that creates free radicals which, on UV irradiation, lead to the formation of new chemical bonds to increase CXL and hence the corneal stiffness.^20^ While the crosslinking reaction rate generally increases with the concentration of riboflavin, riboflavin at the top layer of cornea can block the UV light from reaching the deeper layers of cornea when its concentration is too high. This would limit the mechanical strengthening to only the superficial layers of cornea. Thus, the overall corneal stiffness does not increase monotonically with riboflavin concentration. This phenomenon explains the seemingly surprising results in the measurement of Young's modulus from in vivo experiments (Figure 7). A more effective strengthening upon CXL was observed for Epi‐Off cornea exposed to riboflavin at 0.1% than at 0.5%. Intact corneas treated with 15 min of US exceeded that with 30 min for the enhancement of Young's modulus, despite a higher amount of riboflavin absorbed in the cornea for the longer US treatment (Figure 6). Consistently, the best performer for corneal strengthening in the Epi‐Off groups and the US‐treated groups are similar in the total corneal absorption of riboflavin and riboflavin penetration profiles across the cornea. Notably, the required exposure time to riboflavin solution was reduced by half in the US method. The shortened treatment time may also be viewed as an advantage for potential clinical translation.

At MI = 0.8, we reason that transient cavitation would be dominant, and mechanical damage on the corneal epithelium will be a double‐edged sword. On one hand, cavitation would create portals for a more effective transport of riboflavin. On the other hand, cavitation might create harmful damage to the ocular tissue. Therefore, in the optimization of the US treatment, we aimed to identify the shortest sonication time to attain sufficient riboflavin delivery, such that side effect would be minimized. The corneal barrier to hydrophilic compounds such as riboflavin is located in the first two surface layers of the epithelium, composed of elongated cells connected laterally with tight junctions.^33^

A significant portion of cells in the superficial layers, which were directly exposed to US and drug solution, were disrupted (Figure 9), while the basal cell layer underneath remained mostly normal. The compromised barrier function was evident from the increased riboflavin absorption and its penetration in the corneal stroma (Figures 3 and 6). The pits (or high absorption zones of fluorescence) observed on the corneal surface by visual inspection (Figures 2 and 5) likely corresponded to regions with most severe damage, which were clearly visible for 40 kHz US treatment when MI was at 0.8 and sonication time was at least 15 min. We anticipate that the focal points of epithelial damage were due to the cavitation activity from the application of low‐frequency US, 40 kHz near the ocular surface. Such observations have also been reported in transdermal applications with low‐frequency US. For instance, the increased skin conductivity and drug permeability were supported by the physical pitting effects on the aluminum foil by US operating at 20 and 40 kHz.^34^, ^35^ In addition, Watanabe et al. performed sonophoresis at 270 kHz and observed small pits on skin surface in scanning electron microscopy (SEM) analysis.^36^

When US was applied for 15 min, the damage to the ocular tissue was reversible. The recovery time with the reported method was 3–4 days (Figures 3 and 4), which is shorter than the conventional approach. Although the abrasion of the corneal epithelium and the disruption of tight‐junctions were clear immediately after US, the damage confined to micron‐sized zones was at a much smaller scale than the debridement of the entire corneal epithelium as in the current clinical practice. This could help to reduce the risk of infection in the immediate postoperative period of CXL. Moreover, the histological results (Figure 9) demonstrated that the integrity of the basal cells and basement membrane was preserved under the optimized US treatment conditions, which is critical for the healing of the corneal epithelium and the restoration of the barrier function.

## 5 CONCLUSION

Low‐intensity low‐frequency US assisted with cooling can mediate the delivery of riboflavin into the corneal stroma to increase corneal stiffness by CXL, at a level comparable to the conventional approach that requires complete de‐epithelization. Acoustic cavitation induces temporary disruption of the corneal epithelium and opens portals for the transport of the hydrophilic compound. The mechanical damage to the corneal epithelium is limited to the superficial layers and reversible. The US method, capable of delivering riboflavin with minimum invasiveness, has the potential to improve the CXL treatment for keratoconus.

## AUTHOR CONTRIBUTIONS

**Zhe Sun:** Data curation (lead); formal analysis (lead); investigation (lead); methodology (lead); project administration (equal); visualization (equal); writing – original draft (lead); writing – review and editing (supporting). **Zhiming LI:** Data curation (supporting); formal analysis (supporting); investigation (supporting); methodology (supporting); visualization (supporting); writing – original draft (equal); writing – review and editing (supporting). **Jin Teng Chung:** Conceptualization (equal); data curation (supporting); formal analysis (supporting); funding acquisition (supporting); investigation (equal); methodology (equal); visualization (supporting); writing – original draft (supporting); writing – review and editing (supporting). **Laurence Chi Ming Lau:** Data curation (supporting); formal analysis (supporting); investigation (supporting); methodology (supporting); project administration (supporting); supervision (supporting); visualization (supporting); writing – review and editing (supporting). **Vishal Jhanji:** Formal analysis (supporting); writing – review and editing (supporting). **Ying Chau:** Conceptualization (lead); funding acquisition (lead); project administration (lead); supervision (lead); writing – original draft (supporting); writing – review and editing (lead).

### PEER REVIEW

The peer review history for this article is available at https://publons.com/publon/10.1002/btm2.10442.

## Supporting information


**Appendix S1:** Supporting Information

## Data Availability

The data that support the findings of this study are available from the corresponding author upon reasonable request.
