# Supplementary material for: Low‐intensity low‐frequency ultrasound mediates riboflavin delivery during corneal crosslinking
Source: Bioeng Transl Med. 2022 Nov 25;8(2):e10442. doi: 10.1002/btm2.10442 (PMC10013762; doi:10.1002/btm2.10442)
Supplement: Supplementary file 1 — Appendix S1: Supporting Information [file BTM2-8-e10442-s001.docx]

Supplementary Data

**Low-Intensity Low-Frequency Ultrasound Mediates Riboflavin Delivery during Corneal Crosslinking**

Zhe Sun^1^, Zhiming Li^1^, Jin Teng Chung^1^, Laurence Chi Ming Lau^1^, Vishal Jhanji^2^, Ying Chau^1,*^

^1^ Department of Chemical and Biological Engineering, The Hong Kong University of Science and Technology, Hong Kong SAR, China.

^2^ Department of Ophthalmology, University of Pittsburgh School of Medicine, Pittsburgh, Pennsylvania, USA

*Corresponding author

**Table S1.** Young's modulus (MPa) of porcine corneas for different US treatments under CXL and Non-CXL conditions.

| **Treatment** | **Young’s Modulus, E (MPa)** | | |
| --- | --- | --- | --- |
|  | Non-CXL | CXL | Fold change |
| Naive | 18.88±2.53 | | |
| 0.5% Ribo--Epi-on | 19.75± 2.66 | 19.38±2.33 | 0.98 |
| 0.5% Ribo--40kHz MI=0.2 | 18.92±2.61 | 19.12±3.04 | 1.01 |
| 0.5% Ribo--40kHz MI=0.4 | 18.33±3.05 | 18.66±3.44 | 1.02 |
| 0.5% Ribo--40kHz MI=0.8(10min) | 18.75±3.61 | 19.40±4.48 | 1.03 |
| 0.5% Ribo--40kHz MI=0.8(20min) | 17.72±4.75 | 20.26±3.45 | 1.14 |
| 0.5% Ribo--40kHz MI=0.8(30min) | 18.54±3.13 | 25.27±2.43 | 1.36 |
| 0.5% Ribo--Epi-off | 19.78±3.38 | 26.31±3.66 | 1.33 |

For the Hydrophone Scanning method to calibrate frequency 40 kHz, following calculation was performed to get the intended MIs.

$$I_{S}P=\frac{P_{r}^{2}}{\rho c}$$

where ISP is the spatial peak intensity (W/cm^2^), Pr is the rarefaction peak pressure (Pa), ρ is the density of water at room temperature, which is 1g/cm^3^ and c is the speed of sound in water, 148000 cm/s at room temperature.

Based on its spatial peak intensity, MI can be determined in the following equation,

$$MI=\frac{P_{r}}{\sqrt{f}}$$

where Pr is the rarefaction peak pressure (MPa) and f is the frequency (MHz) of ultrasound. Hence, calibration curves of ISP versus input voltage and MI versus input voltage could be obtained.

**Table S2.** Young's modulus (MPa) of rabbit corneas for different US treatments under CXL and Non-CXL conditions.


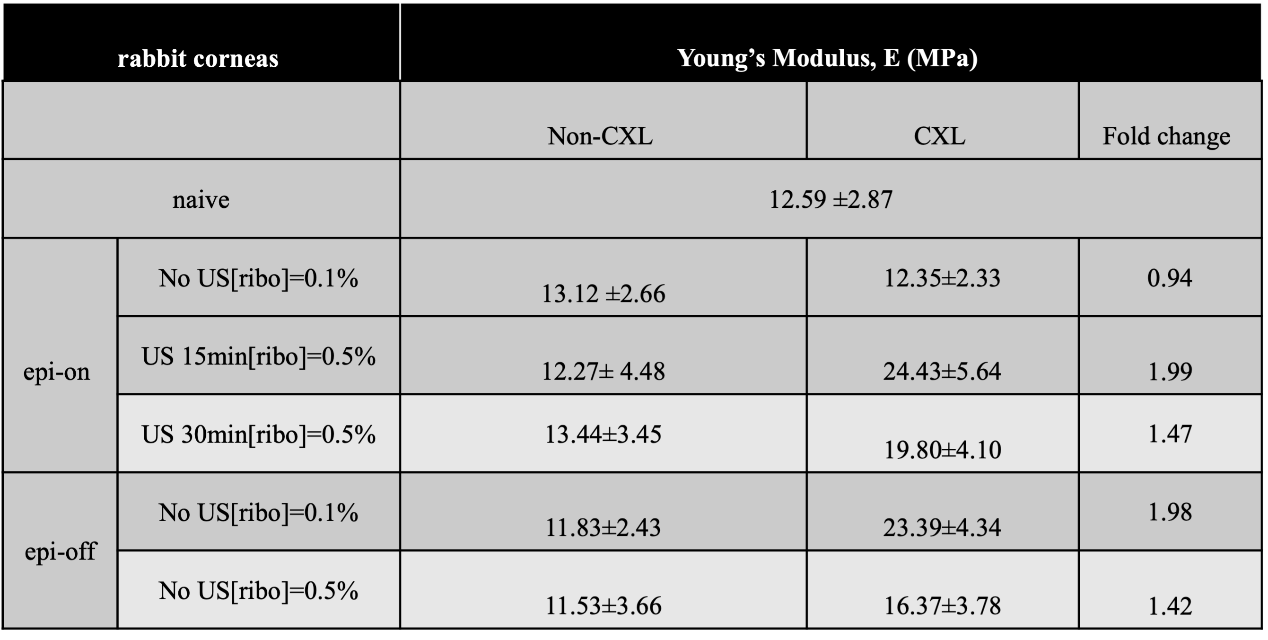


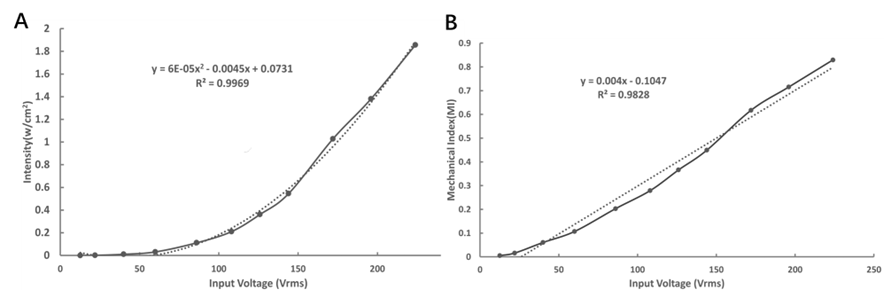


**Figure S1.** The input voltage reationship A) ultrasound intensity B) mechanical index.


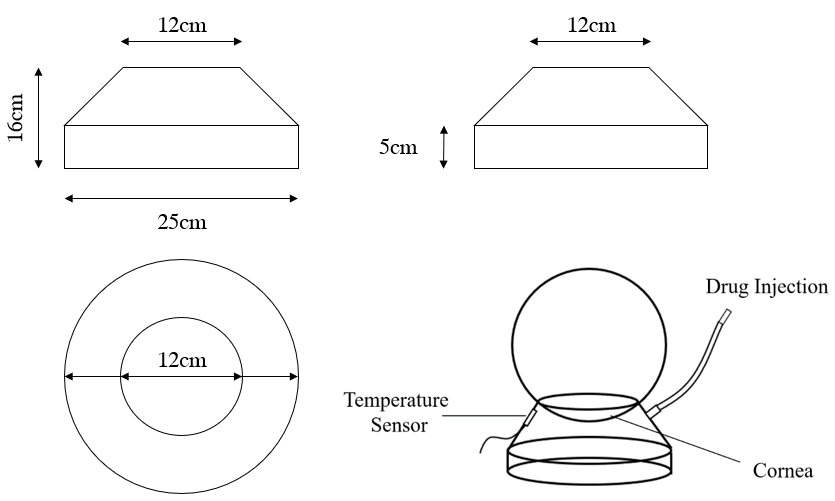


**Figure S2.** The schematic of a drug adaptor configured to be applied to porcine/rabbit cornea for corneal drug delivery.


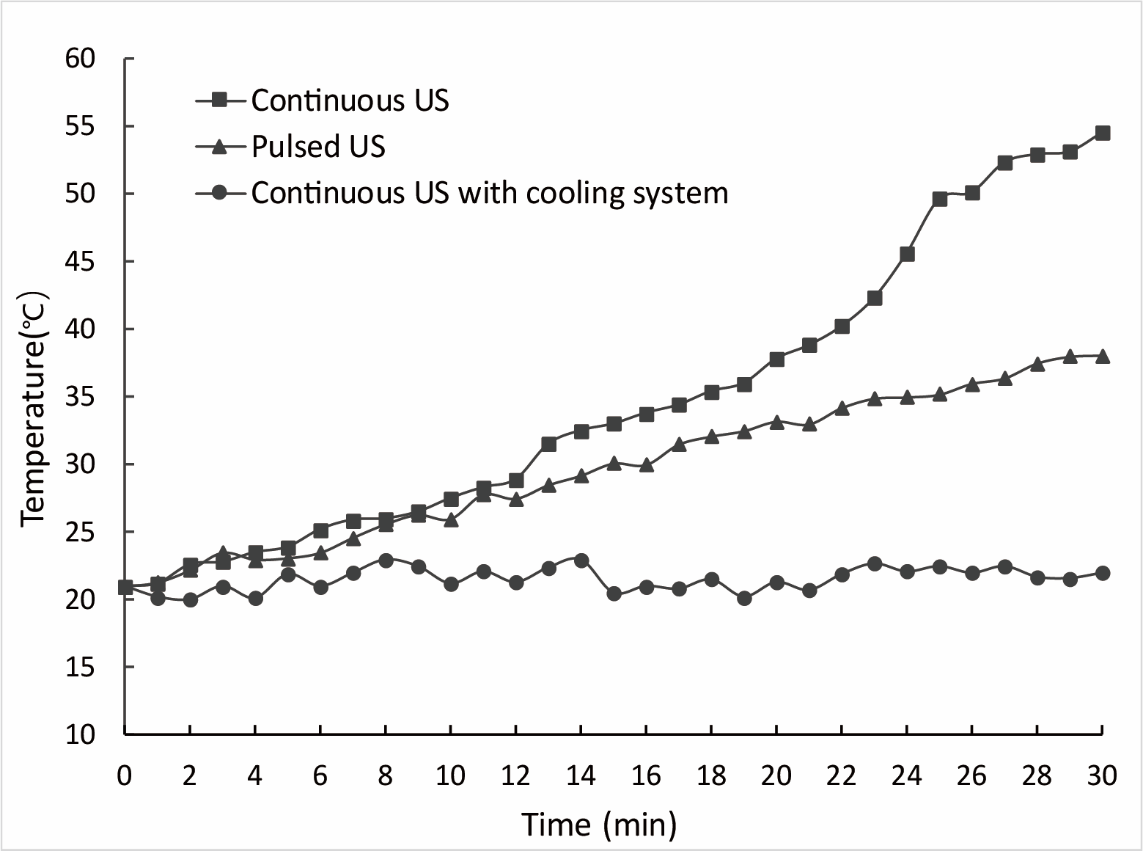


**Figure S3.** The temperature controlled by ultrasound experiment cooling system set up at 40kHz MI=0.8. Change of ex vivo porcine cornea surface temperature after applying continuous ultrasound at 40kHz and MI 0.8; pulsitile ultrasound at 40kHz and MI 0.8 with 30s on/off durations; and continuous ultrasound at 40kHz and MI 0.8 with a collng system.


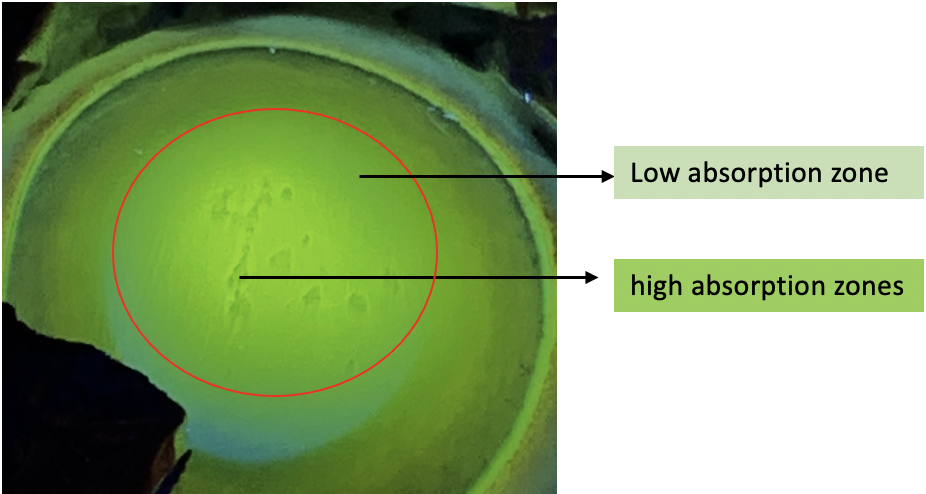


**Figure S4.** The illustration of high and low absorption zone on the ex-vivo porcine corneal surface.

**
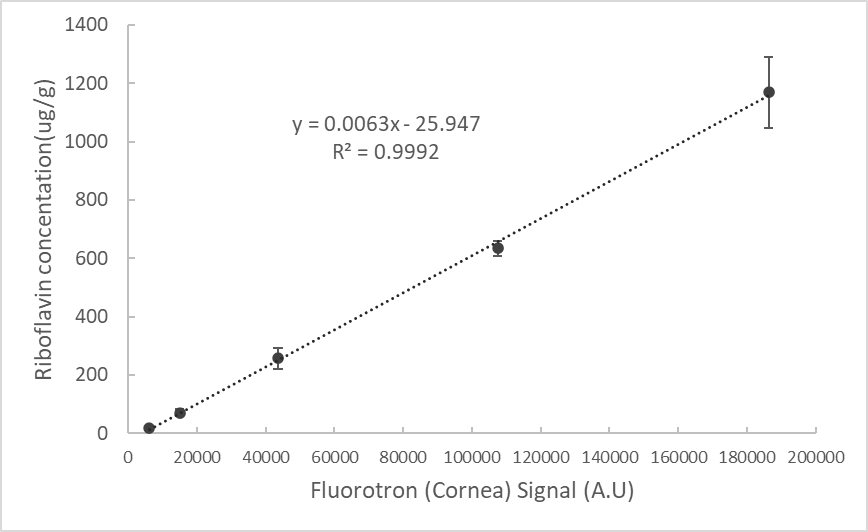
**

**Figure S5.** The correlation curve of riboflavin concentration and fluorescence intensity by cornea of modified Fluorotron. The negative intercept of the fitting curve means that the background noise of Cornea Fluorotron Prototype in the process of scanning the cornea sample.

**
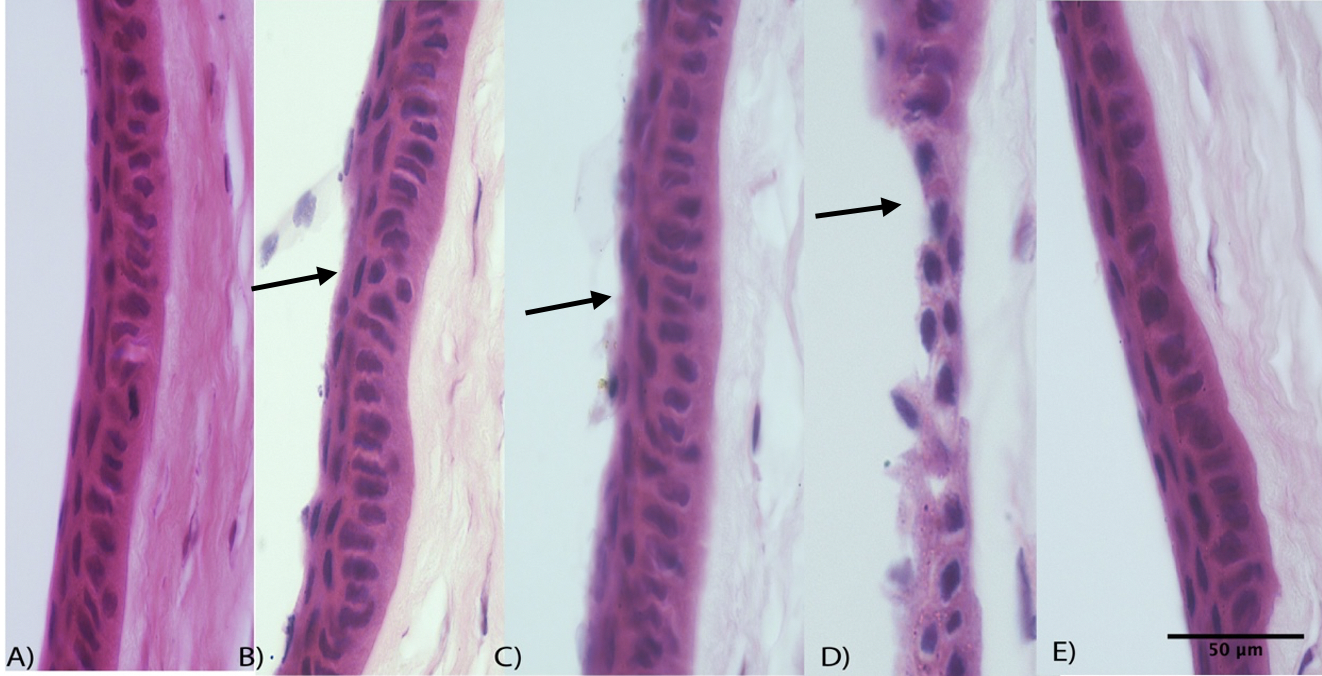
**

**Figure S6.** Histological analysis. Representative Hematoxylin and Eosin histopathology images on 100x magnification from A) native rabbit corneas and B) C) D) rabbit cornea exposed to ultrasound with 40kHz MI 0.8 15min E) rabbit cornea after 4 days until fully recovery (without defect). Black arrows indicate tissue aberrations.
